# Molecular insights: Suppression of EGFR and AKT activation by a small molecule in non-small cell lung cancer

**DOI:** 10.18632/genesandcancer.154

**Published:** 2017-09

**Authors:** Balaji Chandrasekaran, Ashish Tyagi, Arun K. Sharma, Lu Cai, Murali Ankem, Chendil Damodaran

**Affiliations:** ^1^ Department of Urology, University of Louisville, Louisville, KY, USA; ^2^ Department of Pharmacology, Penn State University, Hershey, PA, USA; ^3^ Pediatrics Research Institute, The Department of Pediatrics of the University of Louisville, Louisville, USA

**Keywords:** growth inhibition, EGFR, AKT, lung cancer

## Abstract

Epidermal growth factor receptor (EGFR) activation events and the mammalian target of rampamycin (mTOR) are considered important therapeutic targets in alleviating cancer conditions. The current treatment paradigm has shifted to personalized treatment strategies with tyrosine kinase inhibitors (TKIs) or anaplastic lymphoma kinase (ALK) inhibitors, due to low survival rates in non-small cell lung cancer (NSCLC) in terms of the prevailing platinum-based therapy. In the present study, we examined the anticancer potential of Verrucarin J (VJ), a small molecule, in NSCLC cell lines (H460 and A549). The small molecule significantly inhibited cell growth, proliferation, colony forming ability, and induced apoptosis in both lung cancer cell lines. The inhibitory effects on EGFR (pEGFR –tyr1173) and AKT (pAKT Serine473) signaling, downregulates downstream pro-survival signaling (mTOR and NF-κB) in cancer cell lines. In addition, VJ abrogated invasive and migratory potential of A549 and H460 cells. We also observed a downregulation of mesenchymal markers such as N-cadherin, Slug, β-catenin, and vimentin expression in both cell lines. Our results suggest that VJ inhibited cancer cell growth and could be a potent molecule to inhibit EGFR and AKT signaling in NSCLC.

## INTRODUCTION

Non-small cell lung cancer (NSCLC) is a prevalent malignancy and leading cause of cancer death in both males and females worldwide. In 2016, an estimated 224,390 cases were diagnosed and 158,080 deaths were reported in the US alone [1]. Although targeted therapies have transformed NSCLC treatment regimens, the success in securing a decent survival rate has only been moderate [2]. Novel therapeutic approaches with small molecules tend to have a combined effect on crucial targets, such as EGFR and mTOR.

The epidermal growth factor receptor (EGFR), an ErbB family of receptor tyrosine kinases, is typically known to induce cell proliferation and contributes to transformation of cellular phenotypes by facilitating tumor cell growth and survival [2]. It regulates several signaling pathways in response to EGF, and plays a crucial role in governing cell growth, proliferation, and survival. Overexpression of EGFR is seen in many solid tumors, including NSCLC [3]. Although EGFR is identified as an important antitumor target, therapies against EGFR using small tyrosine kinase inhibitors such as Gefitinib, Lapatinib, and Erlotinib were shown to have limited effectiveness [4]. Studies find that inhibition of EGFR phosphorylation alone is insufficient for generating a potent inhibitory response of cell proliferation in cancer cells [5]. Inhibition of mTOR phosphorylation was demonstrated to deter cell proliferation in NSCLC [6]. Dysregulation of mTOR signaling is reported in several malignancies, including lung cancer [7]. Crosstalk between EGFR and mTOR has been reported to act through AKT signaling pathways.

Several signaling pathways contribute to efficient regulation of cell proliferation and epithelial mesenchymal transition (EMT). AKT activation plays a ubiquitous role in the induction of growth, cell proliferation, survival, invasion, migration, and EMT [8]. Oncogenic activation of AKT in turn activates snail, which binds to E-cadherin promoter and blocks downstream transcriptional events, thereby promoting EMT [9]. Furthermore, AKT signaling has been reported to regulate invasion and metastatic cell properties by regulating β-catenin dependent transcription events [10]. Phosphorylated EGFR activates AKT, which then upregulates mTOR activation. Accumulated evidence indicate that the mTOR pathway is essential for regulation of tumor cell motility, invasion, and cancer metastasis [11]. Therapies simultaneously targeting signaling cascades have shown potent inhibitory effect in human pancreatic, and colon cancer cells [12]. Number of small molecules have been identified to halt progression of lung cancer. More specifically, progress in lung cancer biology has led to the development of small-molecule inhibitors of target proteins involved in proliferation, apoptosis, and angiogenesis. [14].

Verrucarin J (VJ) is a macrocyclic trichothecene and it is characterized by a 12,13-epoxide ring and a 9,10-double bond [13]. Trichothecenes are sesquiterpenoid metabolites largely produced by fungi and species of the plant genus Baccharis, family Asteraceae. It has been shown to exhibit a wide range of biological properties like antiviral, anti-cancer [14], antimalarial and antifungal activities. They affect cells by inhibition of protein, DNA and RNA synthesis, interference with mitochondrial function, affecting cell division and membranes [15]. Verrucarin A, another trichothecene is well studied in prostate, pancreatic and breast cancer [16-18]. However Verrucarin J, an equally potent compound is largely neglected.

We explored how the inhibition of EGFR/AKT/mTOR axis may affect the growth of NSCLC cell lines. Our results suggest that Verrucarin J, a small molecule, effectively suppresses activation of EGFR/AKT/mTOR axis, resulting in growth inhibition and EMT phenotype of NSCLC cell lines. EGF induced EGFR activation and mTOR activation in EGFR-expressing H460 and A549 NSCLC cell lines was inhibited. Our results indicate that VJ is a potential suppressor of EGFR activation, induces apoptosis, and inhibits EGFR/AKT/mTOR mediated-EMT signaling in NSCLC.

## RESULTS

### VJ induced cell growth inhibition in H460 and A549 cells

**Figure 1:**
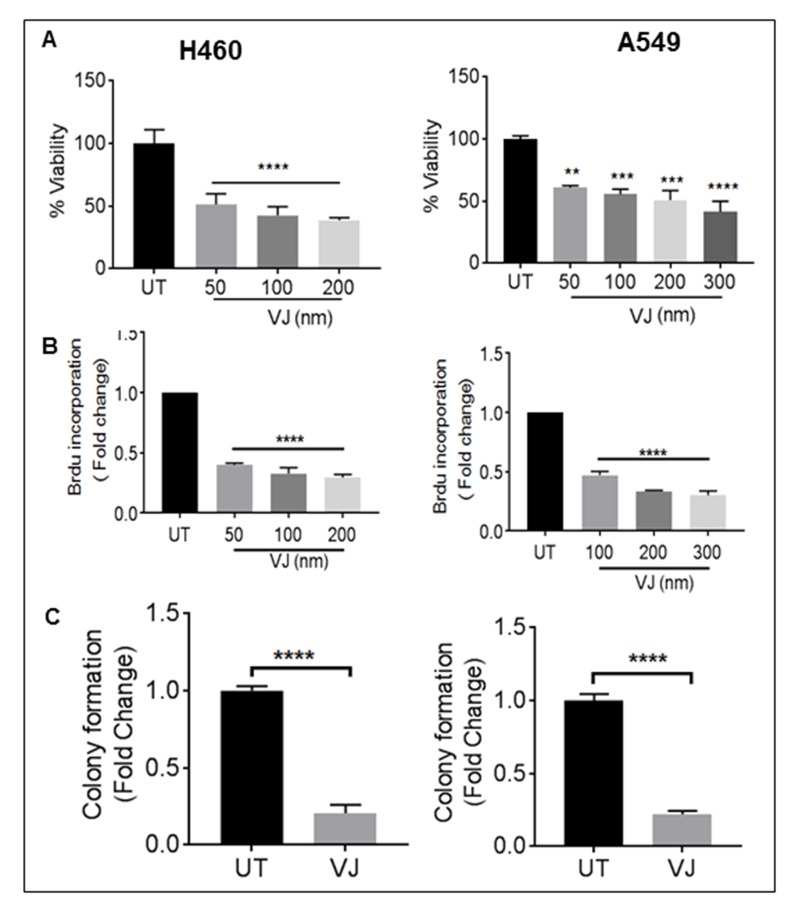
Effect of VJ on cell proliferation **A.** MTT cell proliferation assay of H460 and A549 lung cancer cells treated with 0.1% DMSO (as control) or graded concentration of VJ i.e. 50, 100, 200 nm and 50, 100, 200, 300 nm for 24 hrs resp. **B.** Cell proliferation was assessed by BrdU incorporation-based cell proliferation assay with indicated concentrations of VJ. **C.** Anchorage independent growth was assessed by soft agar colony formation assay in both cancer cell lines. Student's *t*-test was used to calculate statistical significance between VJ treated and untreated at each time point ^*^p < −0.05, ^**^p < 0.01, ^***^p < 0.001.

To understand the biological effects of VJ, we first analyzed cell viability in H460 and A549 cells. Cell proliferation was significantly affected in a dose-dependent manner in both cell lines. The IC_50_ of VJ for H460 ( 50nM at 24h, 25nM at 48h and 5nM at 72h) and A549 (200nM at 24h, 150nM at 48h and 50nM at 72h was calculated (Figure 1A & Supplementary Figure 1). We also performed the Brdu assay, and found a significant reduction in cell viability; this was consistent with our MTT assays (Figure 1B). Further colony forming assays suggested a significant reduction in anchorage independent growth in H460 and A549 cell lines (Figure 1C). These results suggest that H460 cells are more sensitive than A-549 to VJ treatment; however the proliferation is inhibited at the nM concentration in H460 and A549 cells.

### VJ induces apoptosis in H460 and A549 lung cancer cells

**Figure 2:**
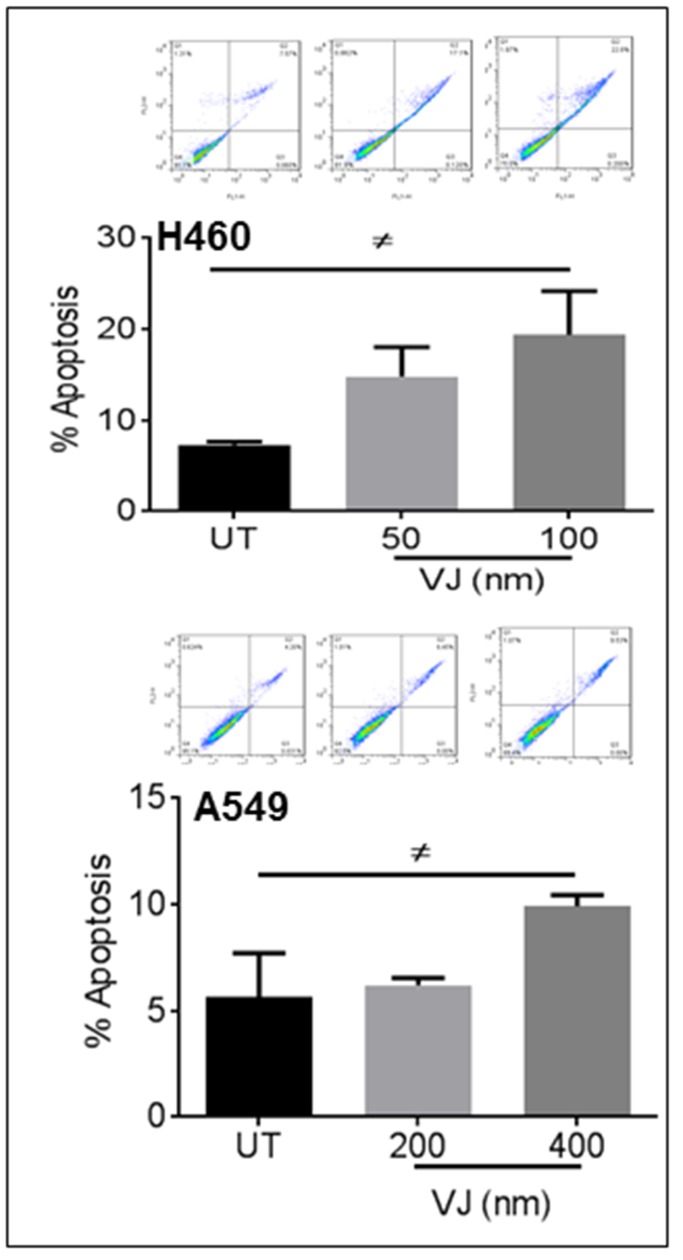
VJ induces apoptosis in H460 and A549 cells Flow cytometry based apoptosis assay was performed using Annexin V –FITC and Propidium Iodide staining for lung cancer cells treated with VJ at indicated concentrations. The percentage of apoptotic H460 and A549 cells were counted from two independent experiments.

**Figure 3:**
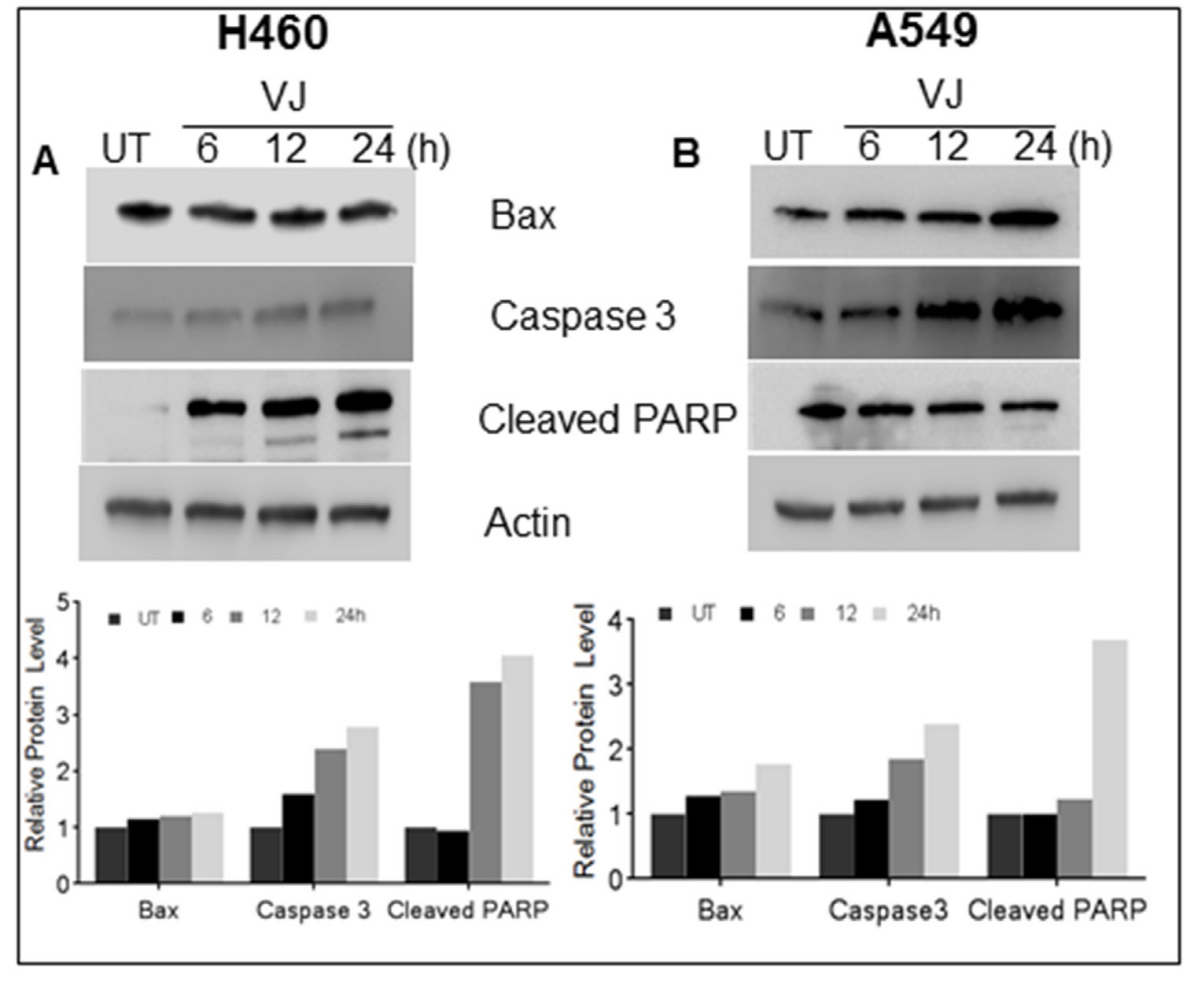
Cells were treated with VJ at time dependent manner and cell lysates quantified for western blot analysis for apoptosis markers i.e. Bax, Caspase3 and Cleaved PARP expression in A) H460 and B) A549 cells β-Actin was used as loading control. The densitometry analyses of bands are expressed in arbitrary units. Analysis was performed using Image Studio Lite 5.2 software.

After confirming the inhibition of cell viability in VJ-treated cells, we ascertained the apoptotic potential of VJ for lung cancer cells. As expected, VJ-induced apoptosis in H460 and A549 cells (Figure 2). Western blot analysis suggested increased expression of Bax, Caspase3, and Cleaved PARP in both H460 (Figure 3A) and A549 (Figure 3B) cells. A significant induction of cleaved PARP were seen at 48h and 72h of VJ treatment on both cell lines (supplementary Figure 2). On the other hand, downregulation of BCl-2 was observed in both cell lines (Figure 6A & 6B). These results suggest that VJ treatment induces cell death through apoptotic pathways in the NSCLC.

### VJ downregulates EGF-induced EGFR signaling in lung cancer

**Figure 4:**
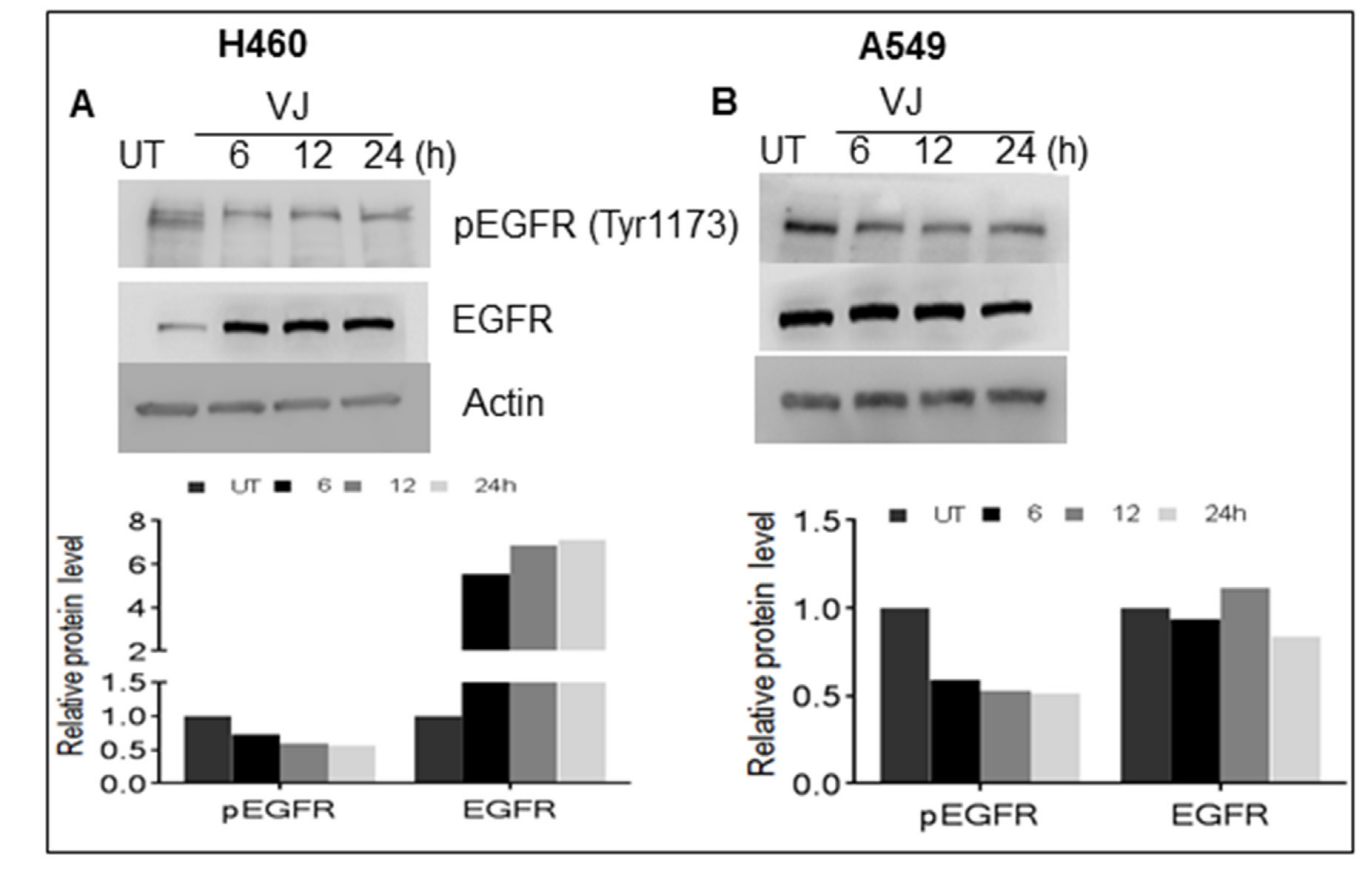
Cells were treated with VJ at IC50 concentrations for 6, 12, 24 hrs. and cell lysates quantified for western blot analysis for effect on basal level of EGFR and phosphorylated EGFR in A. H460 and B. A549 cells β-Actin was used as loading control. The densitometry analyses of bands are expressed in arbitrary units. Analysis was performed using Image Studio Lite 5.2 software.

**Figure 5:**
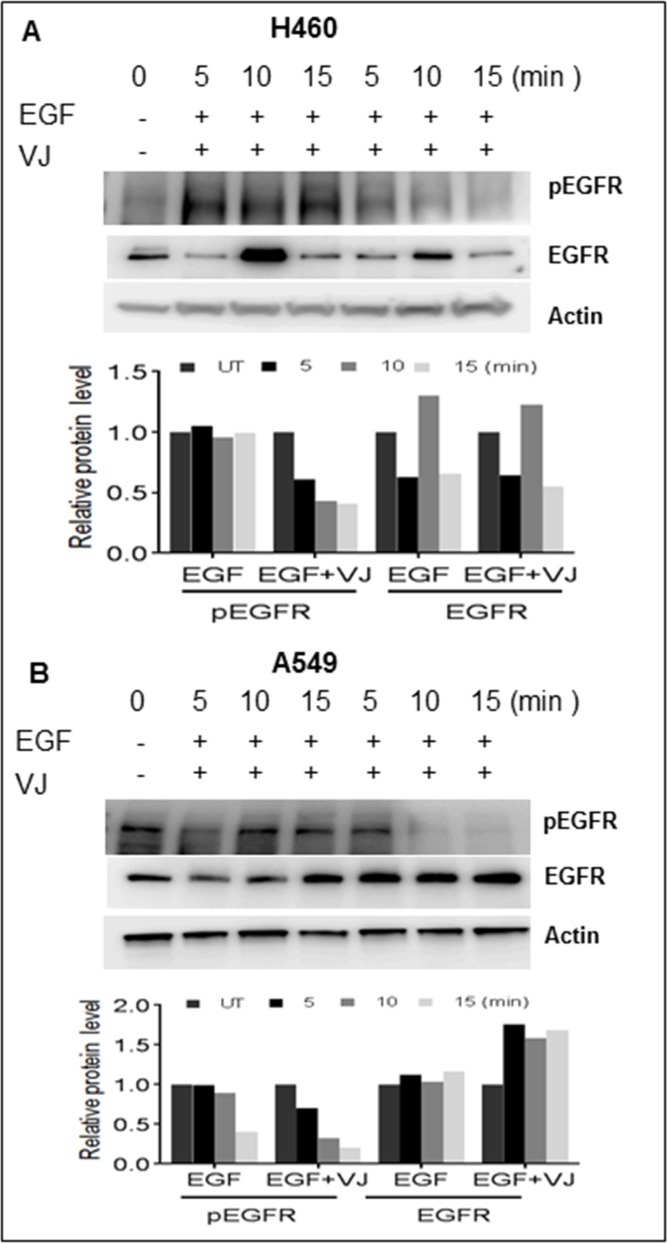
**A.** H460 cells were serum deprived for 24 hrs. and stimulated with 100ng/ml of EGF for up to 15 minutes (Min). Whole cell lysates were subjected to Western blot analysis using pEGFR and EGFR antibodies. **B.** A549 cells were serum deprived for 24 hrs. and stimulated with 100ng/ml of EGF for up to 15 minutes (Min). Whole cell lysates were subjected to Western blot analysis using pEGFR and EGFR antibodies. β-Actin was used as loading control and the densitometry analysis of bands are expressed in arbitrary units.

EGFR is highly expressed in lung cancer, and we were able to determine the effect of VJ treatment on the EGFR activation on both cell lines. A549 cells exhibit higher basal levels of pEGFR (Tyr^1173^) compared to H460 cells (Figure 4A & 4B). Inhibition of pEGFR was seen in H460 and A549, when treated with VJ at respective IC doses (Figure 4A & 4B). Further, we analyzed whether VJ overcome EGF-induced EGFR activation, both cell lines treated with EGF and EGFR activation were measured by western blot analysis. As seen Figure 5A & 5B, VJ abolished pEGFR expression in both H460 and A549 cells suggesting a potent molecule for inhibiting EGFR activation in lung cancer.

### VJ inhibits AKT/mTOR/NF-κB signaling axis in lung cancer cells

**Figure 6:**
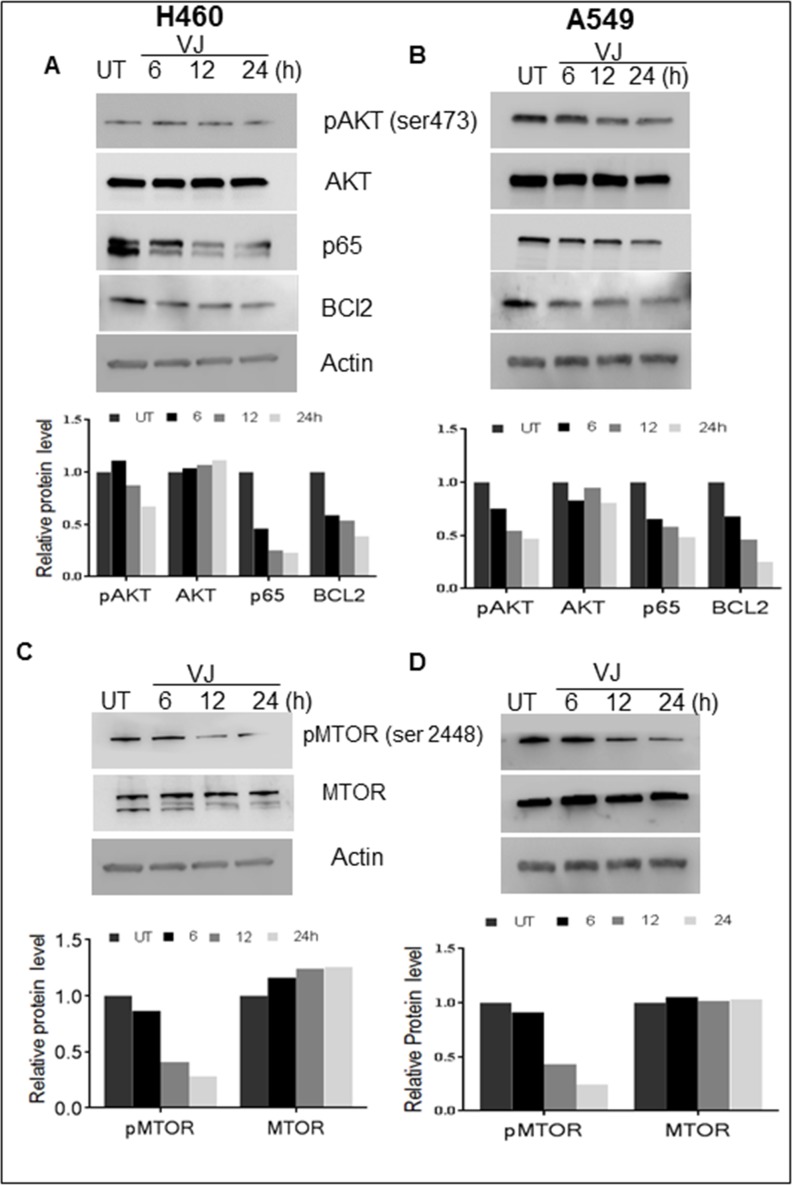
**A.** H460 cells & **B.** A549 Cells were treated with VJ in time dependent manner and cell lysates analyzed via western blot to examine the survival markers phosphorylated AKT, AKT, p65 and BCl2. β-Actin was used as loading control. **C.** & **D.** Western blot analysis of mTOR and phosphorylated mTOR expression in H460 and A549 cells. The densitometry analysis of bands are expressed in arbitrary units.

AKT activation has been implicated in increased cell growth in several cancer types, so we examined whether VJ inhibits the AKT signaling network in lung cancer cells. Significant reduction in pAKT (Ser473) expression was observed 12 h onwards in VJ-treated lung cancer cell lines (Figure 6A & 6B). NF-κB activation by AKT was seen as a major event in activation of pro-survival events. We explored if suppression of AKT activation by VJ affects NF-κB expression in H460 and A549 cells (Figure 6A & 6B). AKT regulates cell survival function and confers resistance to apoptosis by upregulating mTOR signaling axis in cancer cell types. We determined that inhibition of AKT activation resulted in downregulation of pmTOR (ser2448) expression in lung cancer cells (Figure 6C & 6D), showing inhibition of mTOR activation in H460 and A549 cells. These results indicate an inhibitory potential of VJ on pro-survival signaling in H460 and A549 cells.

### VJ inhibits migration and invasion in lung cancer cells

**Figure 7:**
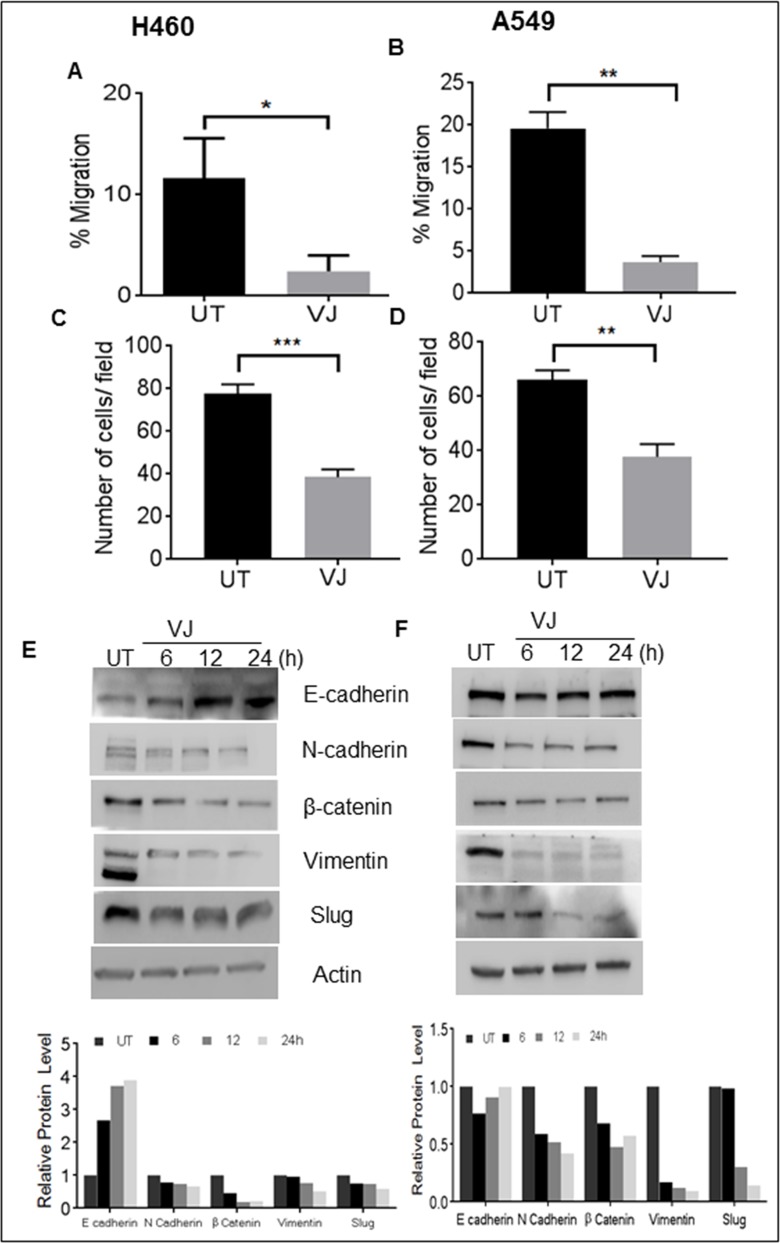
VJ inhibits EMT in H460and A549 lung cancer cells **A**., **B.** Wound healing assay: a wound was created and the H460 and A549 cells were treated with resp. The wound gap was photographed at the same points using the distance between the two edges of wound (ImageJ). **C**., **D.** Transwell invasion assay performed using Boyden chambers. The H460 and A549 invaded cells were stained with crystal violet and counted. **E**., **F.** The H460 and A549 cells were treated with VJ and DMSO (as control), lysates were prepared after 6, 12, 24 hr treatment period and analyzed for E-cadherin, N-cadherin, β-catenin, Vimentin, slug proteins with western blotting. β-Actin was used as loading control and the densitometry analysis of bands are expressed in arbitrary units. Analysis was performed using Image Studio Lite 5.2 software. Student's *t*-test was used to calculate statistical significance between VJ treated and untreated at each time point ^*^p < −0.05, ^**^p < 0.01, ^***^p < 0.001.

Cell migration and invasion are prominent markers of tumor progression and malignancy. Results from wound healing assays clearly demonstrate a significant (P) decrease in migratory potential of H460 and A549 cells in comparison to control (Figure 7A & 7B). As expected, the invasive capacity was also remarkably suppressed in H460 and A549 cells (Figure 7C & 7D).

To assess whether the suppressive effect on migratory and invasive potential are translated at the protein level, we examined the expression of epithelial mesenchymal transition (EMT) related protein markers. VJ treatment upregulated E-cadherin expression and reduced N-cadherin, β-catenin, Vimentin, and Slug expression in a time-dependent manner in both lung cancer cells (Figure 7E & 7F). A significant induction of E-cadherin were observed 48 and 72h treatment of VJ in both A549 and H460 cells (supplementary Figure 2) Based on these results, it is evident that VJ is not only inhibiting cell viability but affecting epithelial to mesenchymal transition capabilities of A549 and H460 cells.

## DISCUSSION

EGFR activation imparts oncogenic potential and is a major signaling cascade in NSCLC [19]. EGFR inhibitors such as erlotinib and gefitinib have become effective options to treat lung cancer; however, it was relatively less successful due to side effects and mutations in EGFR, causing resistance to the treatment which hindered these inhibitors as mainstream agents to treat lung cancer patients. Resistance to treatment due to mutations in EGFR hindered the extensive usage of these agents in lung cancer patients [20]. In our results, we demonstrated abolishing both EGFR and AKT signaling by VJ treatment to suppress the growth of lung cancer cells.

In our studies, VJ inhibits the proliferation of both H460 and A549 cells in a dose- and time-dependent manner: we found, however, that the IC50 concentration of the two cell lines were different. H460 cells were more sensitive to VJ treatment compared to A549. Although both cell lines expressed wild type EGFR [21], the molecular markers such as mutational signatures were different between them. For example the mutation status of genes such as CDKBN2A, K-Ras, STK11, SMARCA4, PI3KCA, and Myc differ in both cell lines [22-25], so we believe these mutations play a significant role in VJ sensitivity in A549 and H460 cell lines. Studies have demonstrated that induction of apoptosis via the mitochondria-initiated death pathway plays an important role in triggering apoptosis in response to various stimuli [26]. Enhanced activation of cleaved PARP (up to 72h) and caspase3 indicate that mitochondrial pathway of apoptosis was involved in VJ induced apoptosis in H460 and A549. The Bcl-2 family proteins are well-characterized as regulators of apoptosis. The multinomial pro-apoptotic members of this family, such as Bax and Bak, act as a gateway for caspase-mediated cell death. The ratio of Bcl-2/Bax is an important determinant of susceptibility to apoptosis [27, 28], which was apparent in our results.

EGF binds with EGFR and stimulates receptor-based dimerization, which is a prerequisite for triggering downstream signaling pathways. EGFR phosphorylation is a key event in the initiation of EGFR-dependent oncogenic signaling cascade. The pEGFR expression was downregulated in both H460 and A549 cells overexpressing EGFR in response to stimulation with EGF. We found similar results with other small molecules of anti-oncogenic potential in NSCLC [29]. In wild type EGFR NSCLC tumors, the response rate (RR) is below 2% in randomized trials with gefitinib, and less than docetaxel [30], a traditional cytotoxic single agent [31]. Constitutive as well as EGFR-independent activation of EGFR downstream targets could be a primary reason for resistance to EGFR-TKIs. Overexpression of AKT, a downstream effector plays a role in maintaining EGFR-resistant phenotype in NSCLC [32]. Among EGFR downstream pathways, the AKT/mTOR pathway is upregulated in most cancers [33-35]. AKT phosphorylation may influence p53-mediated apoptotic pathway [36] and has been correlated with low patient survival [37].

Simultaneous inhibition of different activated pathways in cancer has been demonstrated to be more effective in inducing desirable antiproliferative and apoptotic effects in NSCLCs [38]. These studies indicate that the annulment of EGFR activation alone may not be sufficient for induction of apoptosis in cancer cells [39] and combined targeting of EGFR and EGFR-independent downstream activation events could be an ideal strategy [40]. In the present study, VJ, a small molecule, simultaneously inhibited EGFR, AKT, mTOR activation, and effectively suppressed cell proliferation and promoted induction of apoptosis in lung cancer cells, expressing wild-type EGFR.

In addition, mTOR phosphorylation at the serine 2448 site improves the efficiency of mRNA translation, increases the expression of a series of cell proliferation and differentiation associated proteins, and promotes cancer cell development and progression [41]. Inhibition of AKT signaling alone has been shown to be redundant due to mTOR mediated reactivation of PI3K/AKT signaling cascade [42]. Also, coexpression of EGFR and p-mTOR has been correlated with the differentiation grade of tumors, and suggested to be relevant therapeutic targets in NSCLC [43]. Combined inhibition of EGFR and mTOR exhibited synergistic cytotoxicity in several human tumor cell lines [12]. In our study, we found similar anti-proliferative effects of VJ by combined inhibition on EGFR/AKT/mTOR axis by VJ treatment in both A549 and H460 cell lines. Similar results were reported of how simultaneous inhibition of pAKT expression and mTOR phosphorylation resulted in decreased survival of NSCLC cells and inhibition of tumor growth in vivo [44]. AKT has been shown to activate a major subunit of NF-κB, RelA [45], thus we examined whether inhibition of AKT activation affects NF-κB expression in lung cancer cells. Downregulation of NF-κB expression was observed in both H460 and A549 cells treated with VJ.

The critical step in the development of metastasis and acquisition of resistance to existing cytotoxic and targeted agents, including EGFR-TKIs, is the EMT process. Switching of the epithelial marker E-cadherin to the mesenchymal marker N-cadherin is a characteristic feature of EMT. EGFR/AKT-mediated signaling is involved in different metastatic cancers and its purported role in chemoresistance is well-documented. Inhibition of EGFR activation and pAKT abrogation by VJ can thus reverse cellular mechanisms leading to chemoresistance. A study by Chen et al., [46] demonstrated that lower E-cadherin or higher N-cadherin levels are associated with poor survival outcome in NSCLC patients. VJ notably increased the E-cadherin level (up to 72h) and reduced the N-cadherin level, indicating the inhibition of the EMT process by VJ. Inhibition of tumor invasion or migration is one of the goals in lung cancer, especially in NSCLC patients who die of metastasis instead of the primary lesion [47]. Results of wound healing and the Boyden chamber assays showed an inhibitory effect of VJ on A549 and H460 cell motility and migration, which are two pronounced steps of a metastatic cascade of tumor cells, suggesting the anti-invasive activity of VJ.

In conclusion, results show that combined inhibition of EGFR activation and an AKT signaling cascade has an anti-proliferative effect and inhibits migration and invasion via EGFR/mTOR/AKT axis in EGFR-expressing H460 and A549 cells. EGFR is an important therapeutic target in lung cancer. Co-inhibition of EGFR and AKT phosphorylation by VJ can reverse the chemoresistant fate of platinum resistant cancer cases, but needs further investigation.

## MATERIALS AND METHODS

### Cell lines and reagents

Human lung cancer lines (H460 and A549) were purchased from American Type Culture Collection (Manassas, VA, USA). Verracurin J (VJ) was purchased from Analyticon Discovery. H460 cells were grown in RPMI and A549 cells were grown in F12K (Ham's F-12K Nutrient mixture, Kaighn's Mod.) medium. Cell culture media were supplemented with 10% fetal bovine serum, 1% L-glutamine and antibiotics, while cells were grown in 5% CO2 at 37°C in an incubator.

### Cell viability assay

Cells (1×10^4^ cells/well) were seeded in 96-well plates and grown overnight. After exposure to designated doses of the VJ for 24, 48 and & 72h. We added 20 μL of MTT solution (5 mg/mL in PBS) to each well of 96-well plates. The plates were incubated for four additional h at 37°C. After that, the medium in plates was removed and 200 μL DMSO was added to each well to solubilize the formazan crystals. We determined absorbance with the 96-well microplate reader at 570 nm. Cell proliferation was quantified for H460 and A549 cells using the Bromodeoxyuridine (BrdU) incorporation assay (Cell Signaling, Danvers, MA, USA), according to manufacturer's protocol.

### Soft agar colony formation assay

Colony formation assay was performed to monitor anchorage-independent growth with the CytoSelect™ 96-well In Vitro Tumor Sensitivity Assay Kit (Cell Biolabs, Inc., San Diego, CA, USA). We harvested cells (5 × 103 cells) (H460 and A549) and performed an assay per the manufacturer's instructions. Colonies were stained after 10 days with 0.005% crystal violet and then counted manually.

### Invasion assay

To determine the invasiveness of H460 and A549 cells, we performed invasion assays and evaluated them by employing Boyden chambers equipped with polyethylene terephthalate membranes with 8-μm pores (Corning, Bedford, MA, USA). The cells were cultured in complete medium for 24 h prior to detachment with trypsin EDTA. Subsequently, 5 × 10^4^ cells per chamber were resuspended in culture medium and layered on the Matrigel; after 24 h, the invaded cells were counted with an AMG EVOS Digital Inverted Microscope (Life Technologies, Carlsbad, CA, USA), as described earlier.

### Wound-healing migration assay

The H460 and A549 cells were plated in six-well plates and cultured until they reached confluency. A linear wound was gently created in the monolayer of cells with a 200 μL sterile pipette tip. The cells were washed with PBS/growth medium to remove the detached cells, followed by gently adding fresh medium. The distance between wound gaps was photographed at the same point at 0 and 24 h time points. The wound gaps were measured by Image J software.

### Cell apoptosis detection by annexin V/PI double staining assay

For apoptosis determination, we used the Annexin V/FITC kit as described by the manufacturer instructions (BD Biosciences, San Jose, CA, USA). Briefly, the harvested cells were resuspended in 1X binding buffer at a concentration of 1×10^6^ cells/ml. Moreover, 100 ml of the solution was added with 5 μL of Annexin V-FITC and 5 μL of Propidium Iodide (PI), and then incubated for 15 min at room temperature in the dark. Later, 400 μL of binding buffer was added, and the cells were analyzed by FACS caliber laser flow cytometer. Student's *t*-test was used to calculate statistical significance between VJ treated and untreated at each time point ^*^ P≤ ≠ not significant.

### Protein extraction and western blotting

H460 and A549 cells were treated with vehicle or VJ as indicated time points and cell lysates were prepared with Mammalian Protein Extraction Reagent (Thermo Scientific, Rockford, IL, USA) according to the manufacturer's protocol. The cell lysates subjected to western blotting with specific antibodies against EGFR, Slug, β-catenin, Vimentin, and NF-κB (p65), E-Cadherin, N-Cadherin, and actin (Santa Cruz Biotechnologies, Dallas, TX, USA). AKT, pAKT, pEGFR, BAX, and BCL-2 were purchased from Cell Signaling (Danvers, MA, USA). The positive bands were detected using enhanced chemiluminescence. For EGF experiments, cells were serum-for deprived for 24h and stimulated with EGF (100 ng/ml) alone or treated concurrently with EGF and VJ for 5, 10, and 15 min with the above mentioned concentration.

### Statistical analysis

The data were presented as the mean ± standard deviation (SD or SEM). We determined significant differences between groups using the unpaired Student's t-test and one- way ANOVA. These differences were established at *p* < 0.05. All of the statistical analyses were performed using Prism 7 software (GraphPad Software Inc., La Jolla, CA, USA). Student's *t*-test was used to calculate statistical significance between VJ treated and untreated at each time point ^*^p < −0.05, ^**^p < 0.01, ^***^p< 0.001.

## SUPPLEMENTARY FIGURES



## Abbreviations

NSCLC: non-small cell lung cancer
EGFR: epidermal growth factor receptor
p-EGFR: phosphorylated EGFR
TKIs: Tyrosine Kinase Inhibitors
m-TOR: Mechanistic target of rapamycin
p-mTOR: phosphorylated mTOR
AKT: Ak strain transforming
EMT: Epithelial to mesenchymal transition
NF-κB: Nuclear factor kappa B
