# Supplementary material for: Molecular insights: Suppression of EGFR and AKT activation by a small molecule in non-small cell lung cancer
Source: Genes Cancer. 2017 Sep;8(9-10):713–24. doi: 10.18632/genesandcancer.154 (PMC5724805; doi:10.18632/genesandcancer.154)
Supplement: Supplementary file 1 [file ganc-08-713-s001.pdf]

Molecular insights: Suppression of EGFR and AKT activation by a small molecule in non-small cell lung cancer – Chandrasekaran et al

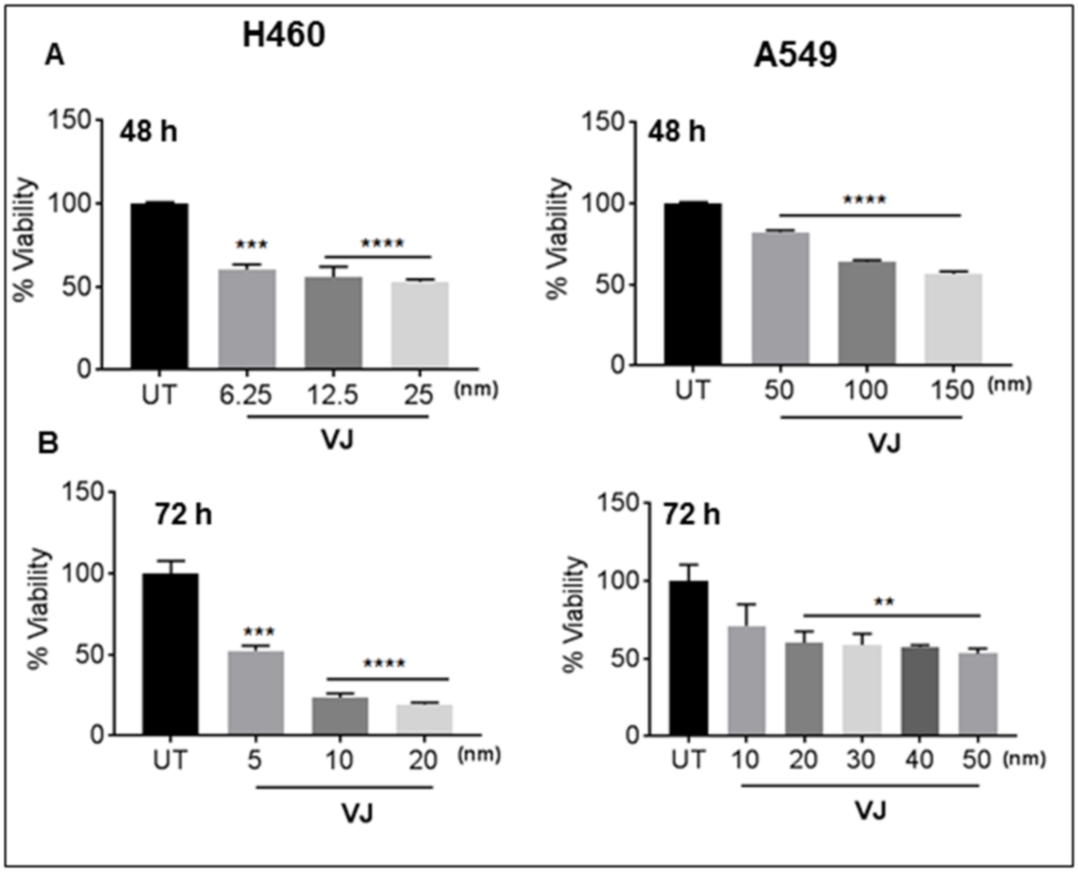

Supplementary Figure 1: VJ inhibits cell proliferation of H460 and A549 cells.

(A) MTT cell proliferation assay of H460 and A549 lung cancer cells treated with 0.1% DMSO (as control) and graded concentrations of VJ (6.25, 12.5, 25nm for H460 and 50, 100 and 150nm for A549) for 48hrs. (B) MTT cell proliferation assay of H460 and A549 lung cancer cells treated with 0.1% DMSO (as control) and graded concentrations of VJ (5, 10 and 20nm for H460 and 10, 20, 30, 40, 50nm for A549) for 72hrs.

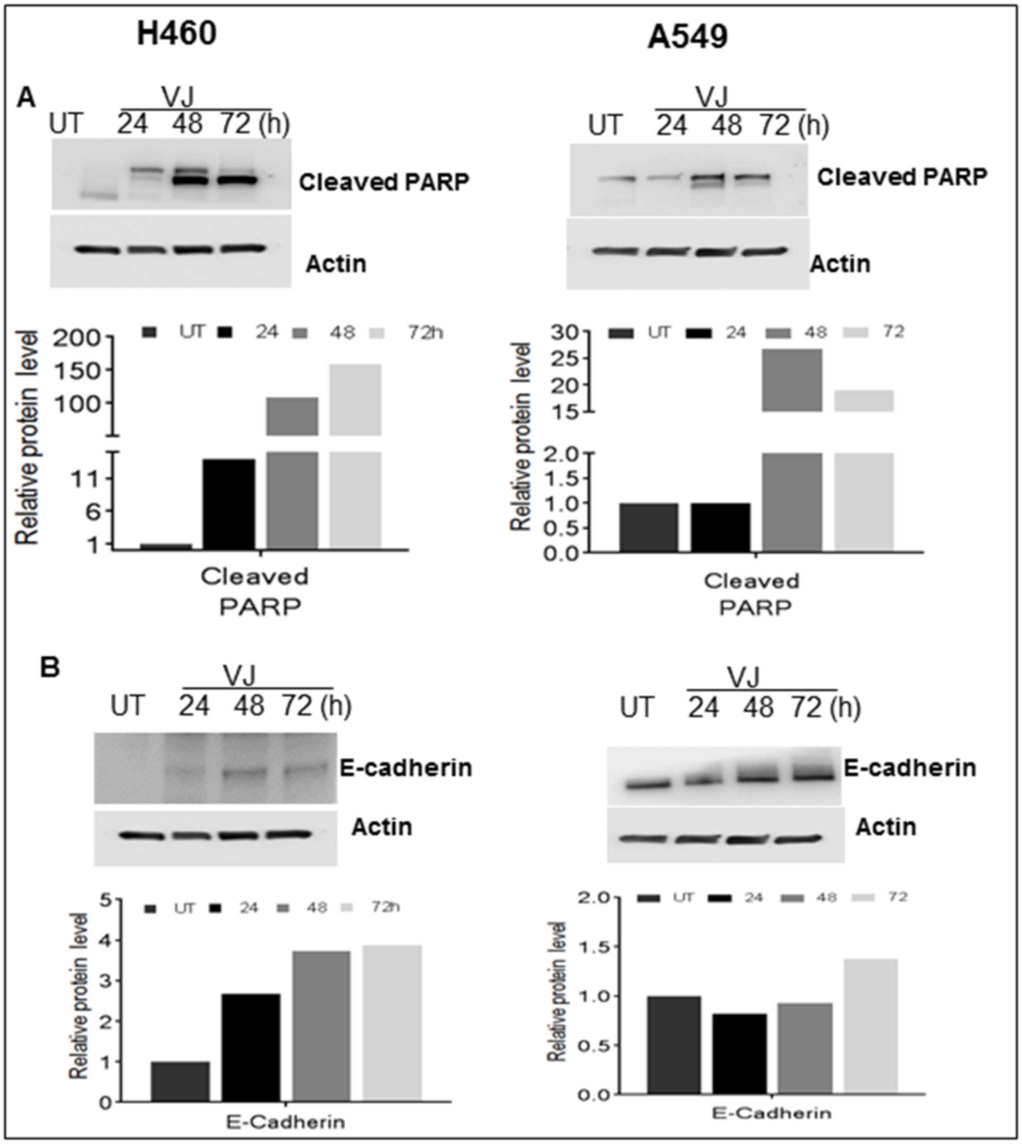

Supplementary Figure 2: (A) H460 cells & (B) A549 Cells were treated with VJ in time dependent manner i.e. for 24, 48 and 72 hrs. Cell lysates analyzed via western blot to examine for Cleaved PARP and E-cadherin expression. β-Actin was used as loading control. The densitometry analyses of bands are expressed in arbitrary units. Analysis was performed using Image Studio Lite 5.2 software.
